# Electronic Properties of Zn_2_V_(1–*x*)_Nb_*x*_N_3_ Alloys to Model Novel Materials for Light-Emitting Diodes

**DOI:** 10.1021/acs.jpclett.3c02242

**Published:** 2023-10-04

**Authors:** Ana-Maria Stratulat, Christian Tantardini, Maryam Azizi, Tariq Altalhi, Sergey V. Levchenko, Boris I. Yakobson

**Affiliations:** † Skolkovo Innovation Center, Skolkovo Institute of Science and Technology, Bolshoy Boulevard 30, Moscow 143026, Russian Federation; ‡ Hylleraas Center, Department of Chemistry, UiT The Arctic University of Norway, PO Box 6050 Langnes, N-9037 Tromsø, Norway; ¶ Department of Materials Science and NanoEngineering, Rice University, Houston, Texas 77005, United States; § Université Catholique de Louvain, Chemin des étoiles 8, bte L07.03.01, B-1348 Louvain-la-Neuve, Belgium; ∥ Chemistry Department, Taif University, Al Hawiyah, Taif 26571, Saudi Arabia

## Abstract

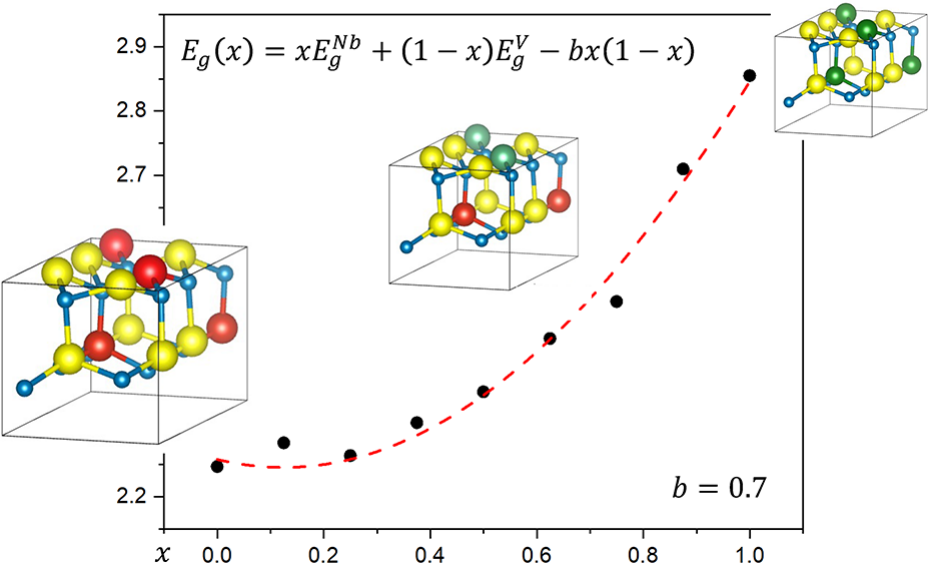

We propose the Zn_2_V_(1–*x*)_Nb_*x*_N_3_ alloy as a new promising material for optoelectronic applications, in particular for light-emitting diodes (LEDs). We perform accurate electronic-structure calculations of the alloy for several concentrations *x* using density-functional theory with meta-GGA exchange–correlation functional TB09. The band gap is found to vary between 2.2 and 2.9 eV with varying V/Nb concentration. This range is suitable for developing bright LEDs with tunable band gap as potential replacements for the more expensive Ga_(1–*x*)_In_(*x*)_N systems. Effects of configurational disorder are taken into account by explicitly considering all possible distributions of the metal ions within the metal sublattice for the chosen supercells. We have evaluated the band gap’s nonlinear behavior (bowing) with variation of V/Nb concentration for two possible scenarios: (i) only the structure with the lowest total energy is present at each concentration and (ii) the structure with minimum band gap is present at each concentration, which corresponds to experimental conditions when also metastable structures are presents. We found that the bowing is about twice larger in the latter case. However, in both cases, the bowing parameter is found to be lower than 1 eV, which is about twice smaller than that in the widely used Ga_(1–*x*)_In_(*x*)_N alloy. Furthermore, we found that both crystal volume changes due to alloying and local effects (atomic relaxation and the V–N/Nb–N bonding difference) have important contributions to the band gap bowing in Zn_2_V_(1–*x*)_Nb_*x*_N_3_.

Light-emitting diodes (LEDs) are used in devices where brightness, low power consumption, and reliability are needed, including automotive, mobile, and display applications. Most bright LEDs are based on group III–V semiconductors or their alloys. Indeed, there are several reported studies of complex semiconductors belonging to the III–V family consisting of elements of group III (B, Al, Ga, and In) and group V (N, P, As, and Sb).^1−13^ In particular, III–N alloys exhibit an ultrawide band gap due to the quantum confinement effect.^14^ Therefore, III–N alloys find applications in blue LEDs and ultraviolet optoelectronic devices^15−17^ and in high-power electronic devices.^17^ Currently, InGa-N alloys are mostly used in industry due to their favorable electronic properties and established synthetic routes. However, these materials and their syntheses are expensive. It is also difficult to produce *p*-type doped GaN, which is needed for blue LEDs. Therefore, there is an active search for semiconductors that could replace InGa-N/GaN materials^1,18^ in electronic devices.

A class of promising nitride compounds for LEDs are polyvalent ternary compounds, which are based on Zn or Mg nitrides with crystal structures derived from parent compounds of wurtzite or rock-salt structures. These nitrides can be combined with GaN and related III–N wurtzite semiconductors, which are amenable to *p*-type doping upon contact formation.^19−21^ Zn_2_PN_3_ (i.e., II–V–N) alloy is characterized by wide gap (higher than 4 eV) and a low electronic effective mass, which makes it suitable for power electronics, where wider gaps allow higher breakdown voltages.^22^ Examples of experimentally synthesized wurtzite materials of the II–V–N family include Zn_2_VN_3_ with a predicted fundamental band gap of 2.23 eV and a direct gap of 2.35 eV.^23^ Zn_2_VN_3_ is a weakly doped *p*-type semiconductor that exhibits broadband room-temperature photoluminescence spanning the range between 2 and 3 eV. Its electronic properties can be tuned over a wide range via isostructural alloying on the cation site, making this a promising material for optoelectronic applications.^23^

The effect of vanadium doping on the electronic and optical properties of various semiconductors is far from trivial. The vanadium impurity in ZnO, according to some authors, increases the band gap and visible transparency, but others have found the opposite behavior.^24−26^ Furthermore, vanadium at high concentration is seen to differently affect the band gap in GaN depending on the magnetic order of vanadium defects: in the case of ferromagnetic ordering, vanadium doping narrows the band gap, while in the case of antiferromagnetic ordering, vanadium increases the band gap.^27^ Another metal dopant that has a nontrivial effect on the band gap of mixed semiconductors is niobium. In ZnS, it causes the band gap reduction in the spin-minority channel.^28^ It has been shown experimentally that Zn_2_NbN_3_ crystallizes in a cation-disordered wurtzite (ZG) structure with an optical band gap of 2.1 eV.^29^ This study presents wurtzite Zn_2_NbN_3_ as a promising new ternary semiconductor for optoelectronic and ferroelectric applications and suggests the possibility of synthesizing quaternary nitride semiconductors by exchanging Nb with V. It is therefore very interesting, both from a practical and fundamental standpoint, to systematically study the electronic structure of Zn_2_V_(1–*x*)_Nb_*x*_N_3_ alloys incorporating Zn (*s*-element) and V and Nb (*d*-elements).

III–N alloys exhibit a nonlinear band gap variation with concentration, called “bowing”.^1,30−50^ The bowing parameter (*b*) determines how far from linear the band gap dependence on alloy composition is. In the general case of a binary alloy α_(1–*x*)_β_*x*_N, *b* is defined as the coefficient of the nonlinear term in the phenomenological expression^51^
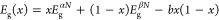
1where *E*_g_^*αN*^ and *E*_g_^*βN*^ are the band gaps for the pure α and β nitrides, respectively. In general, the parameter *b* can also depend on *x*, accounting for higher-order deviations from linearity. Moreover, for each concentration *x* the band gap of an alloy *E*_*g*_(*x*) depends on the atomic configuration *c* (distribution of the metal atoms in the sublattice). This dependence is called an *alloy broadening*. In the presence of broadening, *b* will depend on the abundance of various configurations in the synthesized materials depending on the synthesis method and relative thermodynamic stability of configurations. The parameter *b* indicates whether the band gap is smaller (*b* > 0) or larger (*b* < 0) than the linear combination of end-point band gaps. Hence, to design LEDs with specific optical properties, knowledge of the bowing parameter is of fundamental importance.

A class of promising nitride compounds for LEDs are polyvalent ternary compounds, which are based on Zn or Mg nitrides with crystal structures derived from parent compounds of wurtzite or rock-salt structures. These nitrides can be combined with GaN and related III–N wurtzite semiconductors, which are amenable to *p*-type doping upon contact formation.^19−21^ Zn_2_PN_3_ (i.e., II–V–N) alloy is characterized by a wide gap (higher than 4 eV) and a low electronic effective mass, which makes it suitable for power electronics, where wider gaps allow higher breakdown voltages.^22^ Examples of experimentally synthesized wurtzite materials of II–V–N family include Zn_2_VN_3_ with a predicted fundamental band gap of 2.23 eV and a direct gap of 2.35 eV.^23^ Zn_2_VN_3_ is a weakly doped *p*-type semiconductor that exhibits broadband room-temperature photoluminescence spanning the range between 2 and 3 eV. Its electronic properties can be tuned over a wide range via isostructural alloying on the cation site, making this a promising material for optoelectronic applications.^23^

The effect of vanadium doping on the electronic and optical properties of various semiconductors is far from trivial. The vanadium impurity in ZnO, according to some authors, increases the band gap and visible transparency, but others have found the opposite behavior.^24−26^ Furthermore, vanadium at high concentration is seen to differently affect the band gap in GaN depending on the magnetic order of vanadium defects: in the case of ferromagnetic ordering, vanadium doping narrows the band gap, while in the case of antiferromagnetic ordering, vanadium increases the band gap.^27^ Another metal dopant that has a nontrivial effect on the band gap of mixed semiconductors is niobium. In ZnS, it causes the band gap reduction in the spin-minority channel.^28^ It has been shown experimentally that Zn_2_NbN_3_ crystallizes in a cation-disordered wurtzite (ZG) structure with an optical band gap of 2.1 eV.^29^ This study presents wurtzite Zn_2_NbN_3_ as a promising new ternary semiconductor for optoelectronic and ferroelectric applications and suggests the possibility of synthesizing quaternary nitride semiconductors by exchanging Nb with V. It is therefore very interesting, both from a practical and fundamental standpoint, to systematically study the electronic structure of Zn_2_V_(1–*x*)_Nb_*x*_N_3_ alloys incorporating Zn (*s*-element) and V and Nb (*d*-elements).

In this analysis, we will in addition disentangle various effects causing the nonlinear behavior of the band gap.^52−55^ Changes in alloy composition produce a near-linear volume variation which is well-known as Vegard’s law.^56^ In order to separate the effect of this volume change on the band gap from the electronic effects of varying concentration, we will study the behavior of the band gap at constant volume but varying concentration. Since the disorder plays a role in the broadening of the emission, we will investigate this as well. Furthermore, we will distinguish the nonlinearity of the band gap dependence on concentration for two specific cases: (i) considering only the band gap coming from the structures with lowest total energy at each concentration (as approximate indicator of thermodynamic stability) and (ii) considering only the minimum band gap among all possible structures at each concentration.

Zn_2_VN_3_ and Zn_2_NbN_3_ are seen experimentally in previous works^20,23^ to adopt orthogonal structures. Thus, we have chosen to consider only this polymorph at different concentrations. These alloys contain cation sites occupied by Zn in the oxidation state (+II) and two different possible oxidation states for V or Nb: (i) low oxidation state (+II) or (ii) high oxidation state (+V), depending on the oxidation state of N [(−II) in the first case or (−III) in the second case].

Depending on the oxidation state of V and Nb, the electronic correlation in the localized *d*-orbitals of the transition metal cations can be different and the calculated electronic structure may be more or less sensitive to the approximations in the computational methods. Moreover, Nb/V–N bonds can have a significant covalent character, which decreases the localization of *d*-electrons on the Nb/V ions, further reducing on-center electronic correlation. Indeed, such nitrides were previously shown to be weakly correlated materials as confirmed by combined experimental and theoretical studies.^23,57^ Thus, using on-site models such as the Hubbard *U* model, which is often used to account for strong on-site electronic correlation in ionic transition-metal compounds, is not well-justified for these materials, as it may not fully capture the physics of Nb/V–N bonding. To evaluate the oxidation state, we have performed Bader charge analysis for both extreme concentrations Zn_2_VN_3_ and Zn_2_NbN_3_ within the primitive cell.

The Bader charges for V and Nb are equal to 1.80 and 2.24 au, respectively (see Supporting Information, Table ST.1). Thus, V and Nb are in the low oxidation state (II versus the maximum possible V), and increasing Nb concentration results in a slight increase of the oxidation state of *d*-element present in the structure. At the same time, the oxidation state of nitrogen decreases from −1.38 to −1.52 au. These values seem to indicate that there is a high occupation of *d*-shells of the transition-metal ions. Taking into account the analysis in the earlier work,^23,57^ we conclude that there is indeed a high degree of covalency in V/Nb–N bonding in these materials, which makes them weakly correlated despite the high occupation of the *d*-states.

It is well-known that even for weakly correlated compounds standard local-density and generalized-gradient approximations (GGA) to the DFT functional can significantly underestimate the band gap of semiconductors. To account for this error, we employ TB09 meta-GGA functional as implemented in the electronic-structure software package Abinit.^58−60^ This functional was recently demonstrated to yield accurate band gaps of a wide range of semiconductors,^61^ including nitrides.^1^ Fundamental and direct band gaps calculated with TB09 for Zn_2_VN_3_ are in good agreement with previous hybrid functional (HSE06) calculations (2.21 and 2.28 eV with TB09 versus 2.23 and 2.35 eV with HSE06^23^). For Zn_2_NbN_3_ TB09 direct band gap is 2.87 eV, which is in a very good agreement with the experimental value 3.00 eV.^29^ Considering that the functional works well for the alloy end-points and that the alloying atoms belong to the same group in the periodic table, TB09 will yield accurate band gaps for Zn_2_V_(1–*x*)_Nb_*x*_N_3_, comparable to significantly more computationally expensive hybrid functional HSE06, preventing its systematic use for larger supercells.

To model the new ternary nitride semiconductors, we use an approach in which the formula unit of the three-component semiconductors is doubled, and one of the identical cations in the supercell is replaced by a different cation with the same total charge, resulting in a new four-component compound with a similar crystal structure.^29,62,63^ In this way, we have built 24-atom primitive orthogonal cells of Zn_2_V_(1–*x*)_Nb_*x*_N_3_ alloy with all possible arrangements of V and Nb atoms in the β sublattice, as shown in Figure 4. We consider different volumes for the same concentrations and quantify the alloy broadening in this set of models.

Next, we have generated 1 × 1 × 2 and 1 × 2 × 1 48-atom supercells of Zn_2_V_(1–*x*)_Nb_*x*_N_3_ alloys with all possible atomic arrangements of V and Nb for each concentration *x*. In this study, we do not consider supercells with random atomic positions. In disordered alloys the concept of band dispersion and therefore the difference between direct and fundamental gaps need careful consideration.^64^ However, a detailed analysis of effective band structure using a spectral decomposition^64^ is beyond the scope of this study.

For the fully optimized supercells, the band gap bowing parameter *b* in eq 1 is calculated in this work as a function of concentration 0 < *x* < 1 for each configuration as follows:
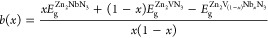
2Subsequently, we have calculated the average bowing parameter *b*_avg_ as follows:
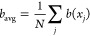
3where the sum is over concentration *j* and *N* is the total number of *b* values.

The obtained fundamental and direct band gaps for the two scenarios (lowest-energy configuration for each concentration and lowest band gap for each concentration) are shown in Figures 1 and 2, respectively. In both cases the band gap varies between 2.2 and 2.9 eV. This is within the range of band gaps for InGa-N alloys (1–3.5 eV).^1^ When only configurations with the lowest total energy are considered (Figures 1a and 2a), the band gap dependence on concentration shows a low bowing of 0.306 eV for fundamental and 0.326 eV for direct band gaps. When metastable structures are present, we observe (see Figures 1b and 2b) about twice larger bowing (0.637 and 0.711 eV for fundamental and direct band gap, respectively). This is explained by generally smaller band gaps for metastable alloy configurations.

**Figure 1:**
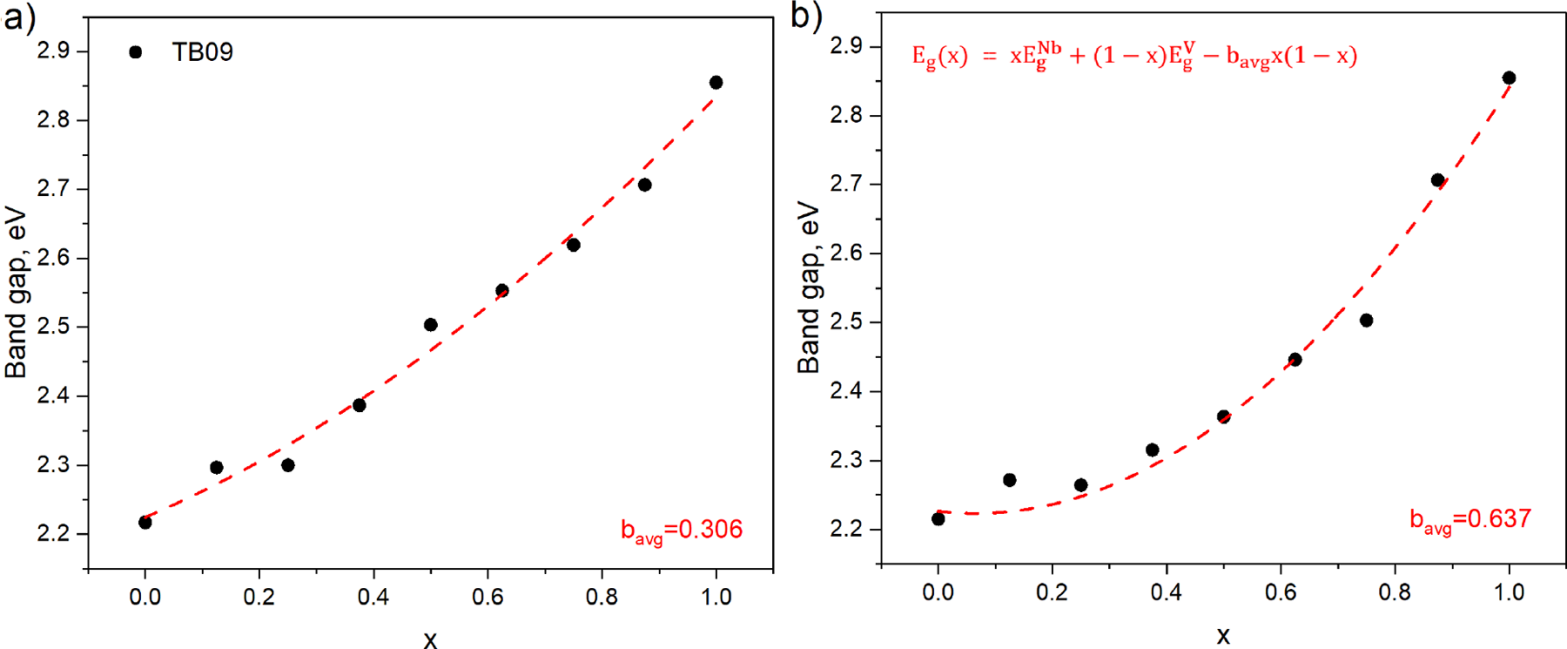
Calculated TB09 fundamental band gap as a function of concentration for (a) the structures with lowest total energy and (b) structures with the lowest band gap among the different V/Nb arrangements in Zn_2_V_(1–*x*)_Nb_*x*_N_3_ 1 × 2 × 1 and 1 × 1 × 2 supercells. The red dashed line in both panels shows the band gap calculated using eq 1 with bowing parameter *b* equal to *b*_avg_ obtained by averaging over the whole range of concentrations.

**Figure 2:**
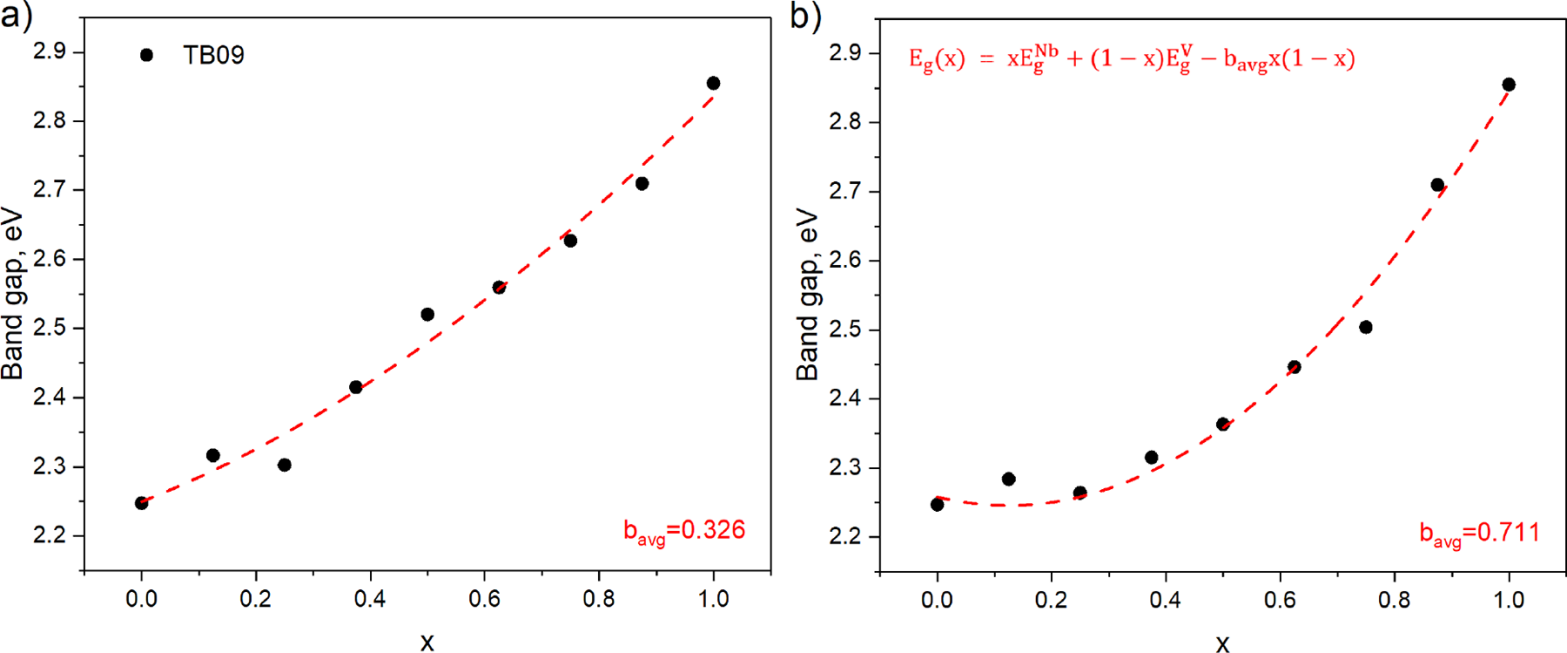
Calculated TB09 direct band gap as a function of concentration for (a) the structures with the lowest total energy and (b) structures with the lowest band gap among the different V/Nb arrangements in Zn_2_V_(1–*x*)_Nb_*x*_N_3_ 1 × 2 × 1 and 1 × 1 × 2 supercells. The red dashed line in both panels shows the band gap calculated using eq 1 with bowing parameter *b* equal to *b*_avg_ obtained by averaging over the whole range of concentrations.

The obtained bowing parameters for Zn_2_V_(1–*x*)_Nb_*x*_N_3_ are a factor of 2 smaller than for Ga_(1–*x*)_In_(*x*)_N alloys, calculated by some of us earlier.^1^ Smaller bowing is beneficial because of a more predictable band gap in synthesized materials in the whole concentration range. This confirms the potential advantage of Zn_2_V_(1–*x*)_Nb_*x*_N_3_ over Ga_(1–*x*)_In_(*x*)_N alloy as alternative materials for LEDs.

To disentangle effects of volume change from local relaxation/electronic effects of alloying on band gap bowing, we have analyzed the fundamental band gap as a function of the concentration *x* for strained crystals by fixing the volume *V*_a_ of 24-atom orthorhombic primitive cells and optimized only the atomic positions for each configuration. In Figure 3a, the volume per atom for all configurations at each concentration *x* in Zn_2_V_(1–*x*)_Nb_*x*_N_3_ alloy supercells (i.e., 1 × 2 × 1 and 1 × 1 × 2) is shown. The increase of Nb concentration for a stoichiometric compound causes a near linear increase in atomic volume, with the root-mean-square deviation (RMSD) from Vegard’s law equal to 0.2 Å/atom. We found that different configurations at the same concentration have very similar equilibrium volumes, barely distinguishable in Figure 3a.

**Figure 3:**
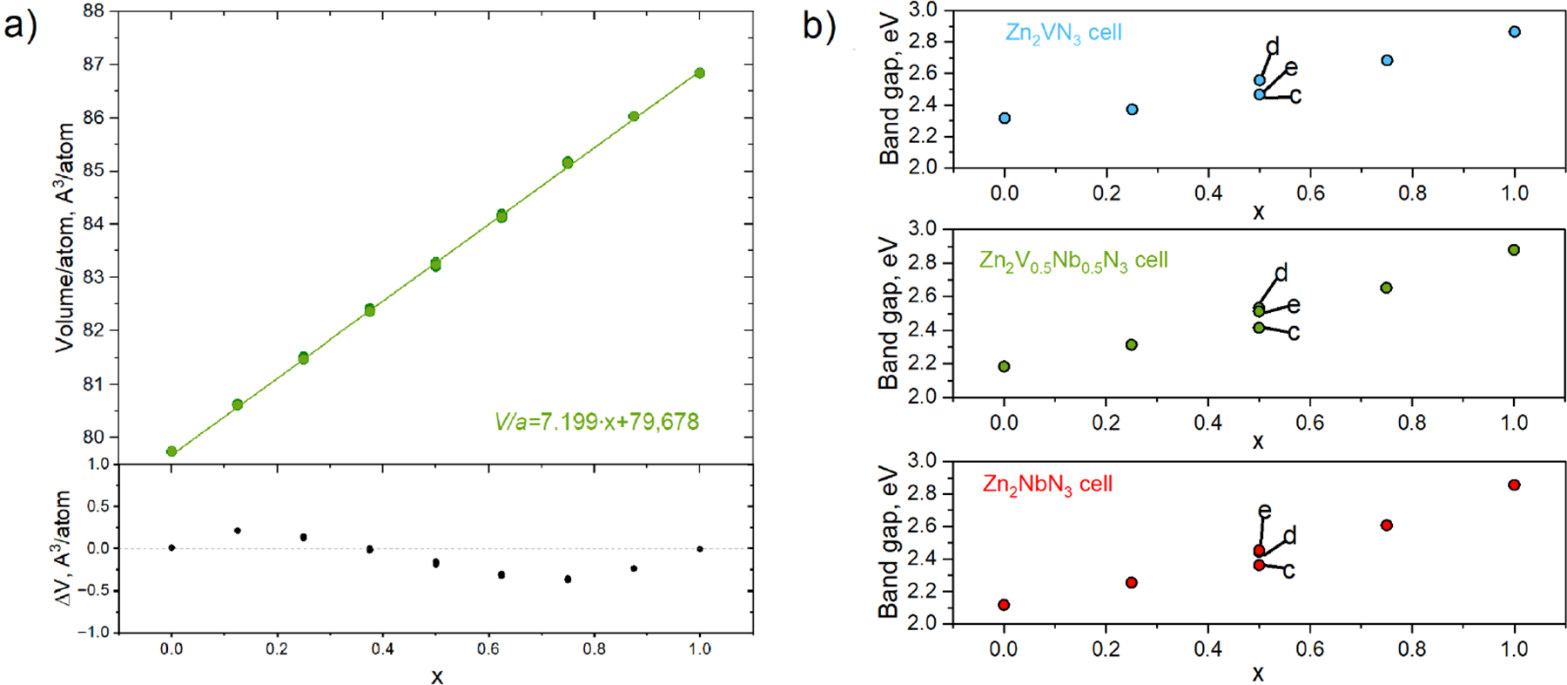
(a) Volume per atom as a function of concentration *x*, calculated by TB09 for orthorhombic 1 × 2 × 1 and 1 × 1 × 2 supercells of Zn_2_V_(1–*x*)_Nb_*x*_N_3_ alloys. (b) Fundamental band gap calculated with TB09 as a function of concentration *x* for fixed volume of 1 × 1 × 1 primitive cells of Zn_2_VN_3_ (cyan), Zn_2_V_(0.5)_Nb_0.5_N_3_ (green), and Zn_2_NbN_3_ (red). The volume is fixed to that of the structure shown in the Figure 4e. Letter labels in panel b correspond to the labels of specific configurations in Figure 4.

Figure 3b shows the fundamental band gap variation with concentration *x* for fixed cell volumes: Zn_2_VN_3_, Zn_2_V_(0.5)_Nb_0.5_N_3_, and Zn_2_NbN_3_. The atoms within each fixed-volume cell are fully relaxed. For Zn_2_V_(0.5)_Nb_0.5_N_3_, the volume was fixed to that of the lowest-energy configuration at this concentration.

Interestingly, although the band gap at fixed stoichiometry decreases with increasing volume and the cell volume increases upon adding Nb, the band gap increases with Nb concentration, even at small Nb concentrations (12.5%). This is explained by the character of the valence band maximum (VBM) and conduction band minimum (CBM), which can be seen in the projected density of states (pDOS) (see Supporting Information Figures SF.1–SF.3). The VBM in these materials is mainly composed of N 2*p* orbitals, while CBM states are *d* states of transition metal atoms. According to the Bader charge analysis (see Supporting Information Table ST.1), each metal atom loses about two electrons, which should go to the N 2*p* band, and transition-metal atoms also form N 2*p*–*d* bonds with predominantly covalent character as showed by the same population of these bands by pDOS (see Supporting Information Figures SF.1–SF.3). Thus, 2/3 of electrons in the N 2*p* band are mainly localized on N anions. However, slightly more electron density is transferred from Nb to N 2*p* relative to V. The resulting electron repulsion in the N 2*p* band is reduced when the cell volume is increased, which results in lowering of the VBM. Although the CBM also lowers, the VBM lowering is more pronounced due to stronger repulsion in the case of Nb-doping.

Figure 4 shows the atomic structures for different concentrations and configurations of Zn_2_V_(1–*x*)_Nb_*x*_N_3_ 24-atom supercells, which were used to calculate band gaps in Figure 3b. The analysis of the relative energies of different configurations of Nb and V at a concentration *x* = 0.5 shows that the structure with Nb and V forming mixed layers (Figure 4c) is the most unstable one. It has the smallest fundamental band gap of 2.42 eV among the three symmetrically nonequivalent configurations in this unit cell. The configurations d (Figure 4d) and e (Figure 4e) have very close fundamental band gaps of 2.510 and 2.514 eV, respectively. The configuration e is the most stable one (see Tables ST.1 and ST.2 in the Supporting Information). The volume of configuration e was chosen for the fixed volume at *x* = 0.5.

**Figure 4:**
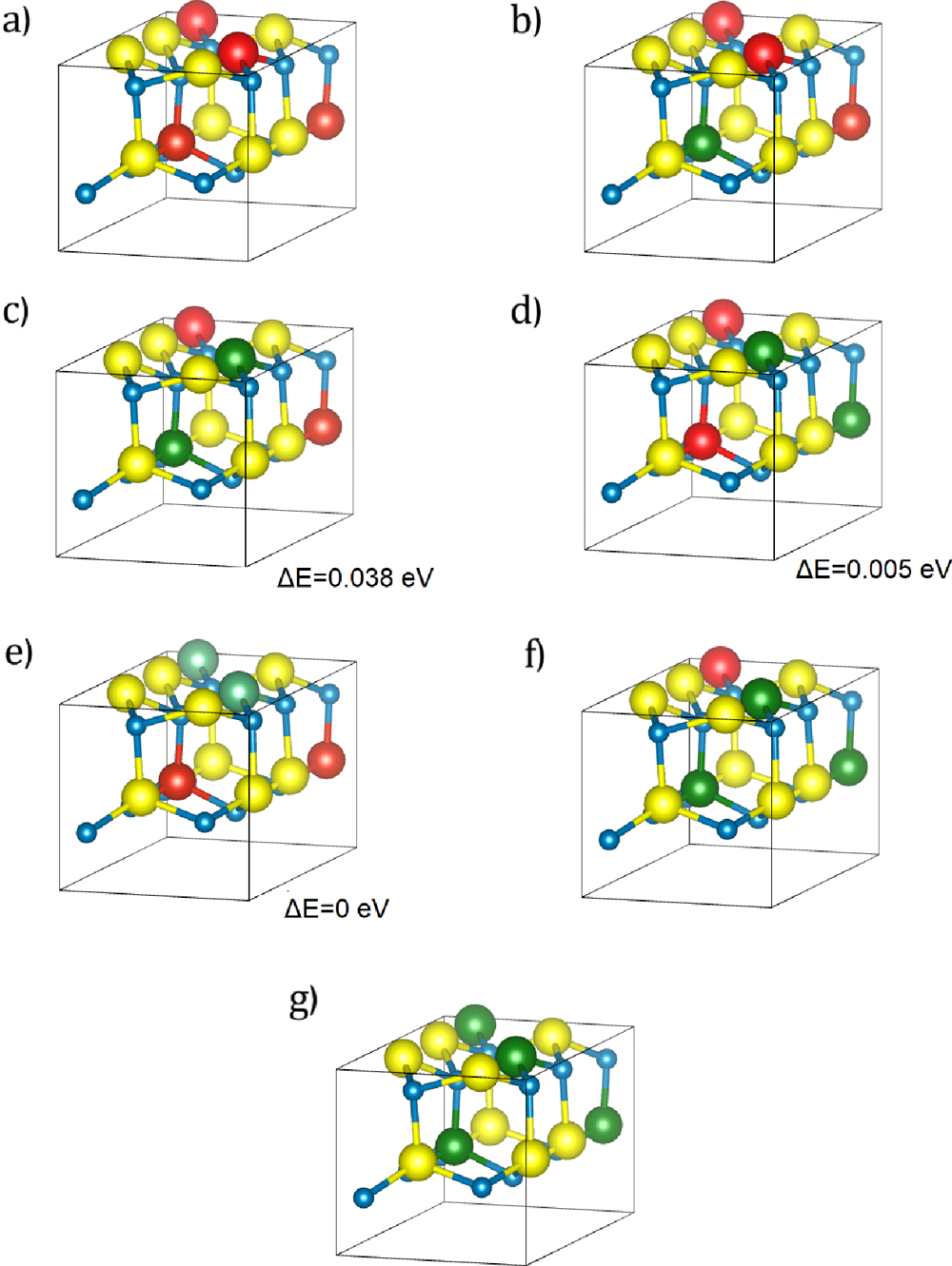
Crystal structure of primitive Zn_2_V_(1–*x*)_Nb_*x*_N_3_ cells: (a) Zn_2_VN_3_, (b) Zn_2_V_0.75_Nb_0.25_N_3_, (c, d, and e) Zn_2_V_0.5_Nb_0.5_N_3_, (f) Zn_2_V_0.25_Nb_0.75_N_3_, (g) Zn_2_NbN_3_. Legend: V, red; Nb, green; Zn, yellow; N, blue. The relative energies Δ*E* with respect to the most stable configuration at *x* = 0.5 are also shown in eV. Legend: V, red; Nb, green; Zn, yellow; N, blue.

The bowing parameters *b*_avg_ calculated from data shown in Figure 3b are summarized in Table 1. When the volume is not fixed for the 24-atom unit cell, the calculated *b*_avg_ is 0.382 eV for the most stable structures (i.e., the band gap of the most stable configuration of Zn_2_V_0.5_Nb_0.5_N_3_ is considered), and 0.495 eV when the configuration of Zn_2_V_0.5_Nb_0.5_N_3_ with the lowest band gap is considered. The data show that fixing the volume does not remove bowing but noticeably reduces it. This means that both volume change and local effects (atomic relaxation and the V–N/Nb-N bonding difference) make important contributions to the band gap bowing.

**Table 1:** Values of *b*_avg_ (in eV) Calculated from Data Shown in Figure 3b

	lowest band gap at *x* = 0.5	most stable structure at *x* = 0.5
Zn_2_VN_3_ volume	0.391	0.269
Zn_2_V_0.5_Nb_0.5_N_3_ volume	0.330	0.172
Zn_2_NbN_3_ volume	0.364	0.242

We calculated the band gap dependence on Nb concentration in Zn_2_V_(1–*x*)_Nb_*x*_N_3_ alloys. A full range of concentrations (0 ≤ *x* ≤ 1) was considered. The band gap is found to vary between 2.2 and 2.9 eV with varying V/Nb concentration. Thus, these novel materials are promising alternatives to the widely used but expensive GaN and InGa-N alloys used for blue LEDs and other optoelectronic applications. The effects of alloy broadening were investigated. The alloy broadening was found to significantly increase the average band gap bowing (from 0.306 to 0.637 eV for fundamental gap, and from 0.326 to 0.711 eV for direct gap) if metastable structures are present in synthesized alloys. In all cases, the bowing parameter is smaller than 1 eV.

The analysis of Bader charges and bonding character revealed that alloying does not change noticeably the nature of bonding in the material. For the whole range of concentrations, VBM is composed mainly of N 2*p* states, while CBM is formed by transition metal *d*-orbitals (3*d* or 4*d* depending on the Nb concentration). Increasing Nb concentration results in near-linear atomic volume increase. Although volume increase is found to reduce the band gap at fixed composition, the band gap increases even at small concentrations of Nb. This is explained by a slightly more ionic character of Nb–N bonds relative to V–N, which results in a more pronounced lowering of the VBM relative to CBM due to the higher electron–electron repulsion in the localized N 2*p* band. Increasing the volume leads to a more pronounced decrease of the energy of the band with larger electronic density. The band gap is indirect for Nb concentrations *x* < 0.5 but becomes direct from *x* = 0.5.

## Supporting Information Available

Computational Details; Bader charges for primitive cells (Table ST.1); concentration, atomic volume, bowing, fundamental, and direct band gaps for the primitive cells (Tables ST.2 and ST.3); concentration and atomic volume for the supercells (Tables ST.4 and ST.5); concentration, bowing, direct, and fundamental band gaps for the supercells (Tables ST.6–ST.13); mixing energies at each concentration for the supercells (Tables ST.14 and ST.15); fundamental band gap at each concentration for primitive cells with fixed volumes (Tables ST.16–ST.18); projected density of states for primitive cells (Figures SF.1–SF.3) (PDF)

## Dedication

The authors would like to dedicate this article to the memory of Alexei Buchachenko.

## References

[ref1] Tantardini C.; Gonze X. Band gap bowing and spectral width of Ga_(1–*x*)_ In_(*x*)_ N alloys for modeling light emitting diodes. Physica B: Condensed Matter 2022, 625, 413481 10.1016/j.physb.2021.413481.

[ref2] Chen Y.; Zhu P.; Wu L.; Chen X.; Lu P. First-principles study on composition-dependent properties of quaternary InP 1- x- y N x Bi y alloys. Modern Physics Letters B 2020, 34, 2050111 10.1142/S0217984920501110.

[ref3] Arora S.; Ahlawat D. S.; Singh D. Estimation of Lattice Constants and Band Gaps of Group-III Nitrides Using Local and Semi Local Functionals. Orient. J. Chem. 2018, 34, 2137 10.13005/ojc/3404055.

[ref4] Abdul Rahim N. A.; Ahmed R.; Haq B. U.; Mohamad M.; Shaari A.; Ali N.; Goumri-Said S. Computational modeling and characterization of X–Bi (X= B, Al, Ga, In) compounds: prospective optoelectronic materials for infrared/near infra applications. Comput. Mater. Sci. 2016, 114, 40–46. 10.1016/j.commatsci.2015.11.043.

[ref5] Bernardini F.; Fiorentini V. Spontaneous versus piezoelectric polarization in III–V nitrides: conceptual aspects and practical consequences. physica status solidi (b) 1999, 216, 391–398. 10.1002/(SICI)1521-3951(199911)216:1<391::AID-PSSB391>3.0.CO;2-K.

[ref6] Luan C.; Lin Z.; Lü Y.; Feng Z.; Zhao J.; Zhou Y.; Yang M. Comparison for the carrier mobility between the III–V nitrides and AlGaAs/GaAs heterostructure field-effect transistors. Journal of Semiconductors 2014, 35, 094007 10.1088/1674-4926/35/9/094007.

[ref7] Fiorentini V.; Bernardini F.; Ambacher O. Evidence for nonlinear macroscopic polarization in III–V nitride alloy heterostructures. Applied physics letters 2002, 80, 1204–1206. 10.1063/1.1448668.

[ref8] Ohta H.; Sztein A.; DenBaars S. P.; Nakamura S. III-V nitride-based thermoelectric device. US 8,692,105 B2, 2014.

[ref9] Freitas J. A.; Zajkac M. Ammonothermal Synthesis and Crystal Growth of Nitrides; Springer, 2021; pp 287–314.

[ref10] Mal I.; Samajdar D. InPNBi/InP heterostructures for optoelectronic applications: A k · p investigation. Materials Science in Semiconductor Processing 2022, 149, 106857 10.1016/j.mssp.2022.106857.

[ref11] Wang S.; Jin T.; Zhao S.; Liang D.; Lu P. Bismuth-Containing Alloys and Nanostructures; Springer, 2019; pp 97–123.

[ref12] Bastos C. M.; Sabino F. P.; Sipahi G. M.; Da Silva J. L. A comprehensive study of g-factors, elastic, structural and electronic properties of III-V semiconductors using hybrid-density functional theory. J. Appl. Phys. 2018, 123, 065702 10.1063/1.5018325.

[ref13] Ahmed R.; Akbarzadeh H.; et al. A first principle study of band structure of III-nitride compounds. Physica B: Condensed Matter 2005, 370, 52–60. 10.1016/j.physb.2005.08.044.

[ref14] Bhasker H.; Thakur V.; Shivaprasad S.; Dhar S. Role of quantum confinement in giving rise to high electron mobility in GaN nanowall networks. Solid State Commun. 2015, 220, 72–76. 10.1016/j.ssc.2015.07.008.

[ref15] Wu K.; Huang S.; Wang W.; Li G. Recent progress in III-nitride nanosheets: properties, materials and applications. Semicond. Sci. Technol. 2021, 36, 123002 10.1088/1361-6641/ac2c26.

[ref16] Yang Y.; Wang W.; Zheng Y.; You J.; Huang S.; Wu K.; Kong D.; Luo Z.; Chen H.; Li G. Defect effect on the performance of nonpolar GaN-based ultraviolet photodetectors. Appl. Phys. Lett. 2021, 118, 053501 10.1063/5.0040110.

[ref17] Sanders N.; Zhang M.; Mengle K.; Qi L.; Kioupakis E. Effect of stacking orientation on the electronic and optical properties of polar 2D III-nitride bilayers. J. Phys. Chem. C 2021, 125, 16837–16842. 10.1021/acs.jpcc.1c04943.

[ref18] Gaffuri P.; Stolyarova E.; Llerena D.; Appert E.; Consonni M.; Robin S.; Consonni V. Potential substitutes for critical materials in white LEDs: Technological challenges and market opportunities. Renewable and Sustainable Energy Reviews 2021, 143, 110869 10.1016/j.rser.2021.110869.

[ref19] Tellekamp M. B.; Miller M. K.; Rice A. D.; Tamboli A. C. Heteroepitaxial ZnGeN_2_ on AlN: Growth, Structure, and Optical Properties. Cryst. Growth Des. 2022, 22, 1270–1275. 10.1021/acs.cgd.1c01232.

[ref20] Zakutayev A.; Bauers S. R.; Lany S. Experimental Synthesis of Theoretically Predicted Multivalent Ternary Nitride Materials. Chem. Mater. 2022, 34, 1418–1438. 10.1021/acs.chemmater.1c03014.

[ref21] Greenaway A. L.; Ke S.; Culman T.; Talley K. R.; Mangum J. S.; Heinselman K. N.; Kingsbury R. S.; Smaha R. W.; Miller E. M.; Persson K. A.; et al. Zinc Titanium Nitride Semiconductor toward Durable Photoelectrochemical Applications. J. Am. Chem. Soc. 2022, 144, 13673–13687. 10.1021/jacs.2c04241. 35857885 PMC9354241

[ref22] Hinuma Y.; Hatakeyama T.; Kumagai Y.; Burton L. A.; Sato H.; Muraba Y.; Iimura S.; Hiramatsu H.; Tanaka I.; Hosono H.; et al. Discovery of earth-abundant nitride semiconductors by computational screening and high-pressure synthesis. Nat. Commun. 2016, 7, 11962 10.1038/ncomms11962. 27325228 PMC4919542

[ref23] Zhuk S.; Kistanov A. A.; Boehme S. C.; Ott N.; La Mattina F.; Stiefel M.; Kovalenko M. V.; Siol S. Synthesis and Characterization of the Ternary Nitride Semiconductor Zn2VN3: Theoretical Prediction, Combinatorial Screening, and Epitaxial Stabilization. Chem. Mater. 2021, 33, 9306–9316. 10.1021/acs.chemmater.1c03025.

[ref24] Tahir N.; Hussain S.; Usman M.; Hasanain S.; Mumtaz A. Effect of vanadium doping on structural, magnetic and optical properties of ZnO nanoparticles. Appl. Surf. Sci. 2009, 255, 8506–8510. 10.1016/j.apsusc.2009.06.003.

[ref25] Ftouhi H.; El Jouad Z.; Jbilou M.; Diani M.; Addou M. Study of microstructural, morphological and optical properties of sprayed vanadium doped ZnO nanoparticles. European Physical Journal Applied Physics 2019, 87, 10301 10.1051/epjap/2019190111.

[ref26] Mhamdi A.; Boukhachem A.; Madani M.; Lachheb H.; Boubaker K.; Amlouk A.; Amlouk M. Study of vanadium doping effects on structural, opto-thermal and optical properties of sprayed ZnO semiconductor layers. Optik-International Journal for Light and Electron Optics 2013, 124, 3764–3770. 10.1016/j.ijleo.2012.11.074.

[ref27] Khan M. S.; Ikram M.; Shi L.-J.; Zou B.; Ullah H.; Khan M. Y. Computational insights into optoelectronic and magnetic properties of V (III)-doped GaN. J. Solid State Chem. 2021, 304, 122606 10.1016/j.jssc.2021.122606.

[ref28] Asfia M. B.; Rashid M. A. First-Principles Study of Half Metallic Ferromagnetic and Optical Properties of Nb Doped Cubic ZnS using TB-mBJ Approximation. Dhaka University Journal of Science 2022, 194–201. 10.3329/dujs.v69i3.60030.

[ref29] Zakutayev A. Synthesis of Zn_2_NbN_3_ ternary nitride semiconductor with wurtzite-derived crystal structure. J. Phys.: Condens. Matter 2021, 33, 354003 10.1088/1361-648X/abfab3. 33887709

[ref30] César M.; Ke Y.; Ji W.; Guo H.; Mi Z. Band gap of In_*x*_Ga_1–*x*_N: A first principles analysis. Appl. Phys. Lett. 2011, 98, 202107 10.1063/1.3592573.

[ref31] Gorczyca I.; Suski T.; Christensen N. E.; Svane A. Band gap bowing in quaternary nitride semiconducting alloys. Appl. Phys. Lett. 2011, 98, 241905 10.1063/1.3597795.

[ref32] Cui Y.; Lee S.; Freysoldt C.; Neugebauer J. Role of biaxial strain and microscopic ordering for structural and electronic properties In_(*x*)_Ga_(1–*x*)_N. Phys. Rev. B 2015, 92, 085204 10.1103/PhysRevB.92.085204.

[ref33] Pugh S. K.; Dugdale D. J.; Brand S.; Abram R. A. Band-gap and k.p. parameters for GaAlN and GaInN alloys. J. Appl. Phys. 1999, 86, 3768 10.1063/1.371285.

[ref34] Wright A. F.; Nelson J. S. Consistent structural properties for AlN, GaN, and InN. Phys. Rev. B 1995, 51, 7866 10.1103/PhysRevB.51.7866. 9977372

[ref35] McCluskey M. D.; Van de Walle C. G.; Master C. P.; Romano L. T.; Johnson N. M. Large band gap bowing of alloys In_(*x*)_Ga_(1–*x*)_N. Appl. Phys. Lett. 1998, 72, 2725 10.1063/1.121072.

[ref36] McCluskey M. D.; Van de Walle C. G.; Romano L. T.; Krusor B. S.; Johnson N. M. Effect of composition on the band gap of strained In_(*x*)_Ga_(1–*x*)_N alloys. J. Appl. Phys. 2003, 93, 4340 10.1063/1.1560563.

[ref37] Bergmann M. J.; Casey H. C. Optical-field calculations for lossy multiple-layer Al_(*x*)_Ga_(1–*x*)_/In_(*x*)_Ga_(1–*x*)_N laser diodes. J. Appl. Phys. 1998, 84, 1196 10.1063/1.368185.

[ref38] Martin R. W.; Edwards P. R.; Hernandez S.; Wang K.; Fernandez-Torrente I.; Kurouchi M.; Nanishi Y.; O’Donnell K. P. The composition dependence of the optical properties of InN-rich InGaN grown by MBE. MRS Online Proceedings Library 2004, 831, 479–484. 10.1557/PROC-831-E3.6.

[ref39] Stepanov S.; Wang W. N.; Yavich B. S.; Bougrov V.; Rebane Y. T.; Shreter Y. G. Influence of Poisson’s ratio uncertainty on calculations of the bowing parameter for strained InGaN layers. Materials Research Society Internet Journal of Nitride Semiconductor Research 2001, 6, E6 10.1557/S1092578300000181.

[ref40] Wu J.; Walukiewicz W.; Shan W.; Yu K. M.; Ager J. W. III; Haller E. E.; Lu H.; Schaff W. J. Effects of the narrow band gap on the properties of InN. Phys. Rev. B 2002, 66, 201403 10.1103/PhysRevB.66.201403.

[ref41] Wu J.; Walukiewicz W.; Yu K. M.; Ager J. W. III; Haller E. E.; Lu H.; Schaff W. J. Small bandgap bowing in In_(1–*x*)_Ga_(*x*)_N alloys. Appl. Phys. Lett. 2002, 80, 4741 10.1063/1.1489481.

[ref42] Pereira S.; Correia M. R.; Monteiro T.; Pereira E.; Alves E.; Sequeira A. D.; Franco N. Structural and optical properties of InGaN/ GaN layers close to the critical layer thickness. Appl. Phys. Lett. 2001, 78, 2137 10.1063/1.1358368.

[ref43] Shan W.; Walukiewicz W.; Haller E. E.; Little B. D.; Song J. J.; McCluskey M. D.; Johnson N. M.; Feng Z. C.; Schurman M.; Stall R. A. Optical properties of In_(*x*)_Ga_(1–*x*)_N alloys grown by metalorganic chemical vapor deposition. J. Appl. Phys. 1998, 84, 4452 10.1063/1.368669.

[ref44] Davydov V. Y.; Klochikhin A. A.; Seisyan R. P.; Emtsev V. V.; Ivanov S. V.; Bechstedt F.; Furthmüller J.; Harima H.; Mudryi A.; Aderhold J.; Semchinova O.; Graul J. Absorption and Emission of Hexagonal InN. Evidence of Narrow Fundamental Band Gap. Phys. Status Solidi B 2002, 229, r1 10.1002/1521-3951(200202)229:3<R1::AID-PSSB99991>3.0.CO;2-O.

[ref45] Takeuchi T.; Takeuchi H.; Sota S.; Sakai H.; Amano H.; Akasaki I. Optical Properties of Strained AlGaN and GaInN on GaN. Jpn. J. Appl. Phys. Part 2 1997, 36, L177 10.1143/JJAP.36.L177.

[ref46] Scholz F.; Off J.; Sohmer A.; Syganow V.; Dornen A.; Ambacher O. MOVPE of GaInN heterostructures and quantum wells. J. Cryst. Growth 1998, 189-190, 8–12. 10.1016/S0022-0248(98)00146-8.

[ref47] Wetzel C.; Takeuchi T.; Yamaguchi S.; Katoh H.; Amano H.; Akasaki I. Optical band gap in Ga_(1–*x*)_In_(*x*)_N (0 < *x* < 0.2) on GaN by photoreflection spectroscopy. Appl. Phys. Lett. 1998, 73, 1994 10.1063/1.122346.

[ref48] Kim M.-H.; Cho J.-K.; Lee I.-H.; Park S.-J. Metalorganic Molecular Beam Epitaxy of InGaN Layers and Their Optical Properties. physica status solidi (a) 1999, 176, 269 10.1002/(SICI)1521-396X(199911)176:1<269::AID-PSSA269>3.0.CO;2-2.

[ref49] Alam S. N.; Zubialevich V. Z.; Ghafary B.; Parbrook P. J. Band gap and refractive index estimates of InAlN and related nitrides across their full composition ranges. Sci. Rep. 2020, 10, 16205 10.1038/s41598-020-73160-7. 33004917 PMC7530746

[ref50] Schygulla P.; Fuß-Kailuweit P.; Höhn O.; Dimroth F. Determination of the complex refractive index of compound semiconductor alloys for optical device modelling. J. Phys. D: Appl. Phys. 2020, 53, 495104 10.1088/1361-6463/abb270.

[ref51] Sandu T.; Iftimie R. I. Bandgaps and band bowing in semiconductor alloys. Solid State Commun. 2010, 150, 888–892. 10.1016/j.ssc.2010.01.046.

[ref52] Magri R.; Froyen S.; Zunger A. Electronic structure and density of states of the random Al 0.5 Ga 0.5 As, GaAs 0.5 P 0.5, and Ga 0.5 In 0.5 As semiconductor alloys. Phys. Rev. B 1991, 44, 7947 10.1103/PhysRevB.44.7947. 9998726

[ref53] Bernard J. E.; Zunger A. Electronic structure of ZnS, ZnSe, ZnTe, and their pseudobinary alloys. Phys. Rev. B 1987, 36, 3199 10.1103/PhysRevB.36.3199. 9943231

[ref54] Zunger A.; Jaffe J. Structural origin of optical bowing in semiconductor alloys. Phys. Rev. Lett. 1983, 51, 662 10.1103/PhysRevLett.51.662.

[ref55] Schnohr C. S. Compound semiconductor alloys: From atomic-scale structure to bandgap bowing. Applied Physics Reviews 2015, 2, 031304 10.1063/1.4930002.

[ref56] Cahn R. W.; Haasen P. Physical metallurgy; Elsevier, 1996; Vol. 1.

[ref57] Kistanov A. A.; Shcherbinin S. A.; Korznikova E. A.; Prezhdo O. V. Prediction and Characterization of Two-Dimensional Zn2VN3. J. Phys. Chem. Lett. 2023, 14, 1148–1155. 10.1021/acs.jpclett.2c03796. 36705575

[ref58] Gonze X.; et al. Recent developments in the ABINIT software package. Comput. Phys. Commun. 2016, 205, 106–131. 10.1016/j.cpc.2016.04.003.

[ref59] Gonze X.; et al. The Abinit project: Impact, environment and recent developments. Comput. Phys. Commun. 2020, 248, 107042 10.1016/j.cpc.2019.107042.

[ref60] Romero A. H.; et al. ABINIT: Overview, and focus on selected capabilities. J. Chem. Phys. 2020, 152, 124102 10.1063/1.5144261. 32241118

[ref61] Waroquiers D.; Lherbier A.; Miglio A.; Stankovski M.; Poncé S.; Oliveira M. J. T.; Giantomassi M.; Rignanese G.-M.; Gonze X. Band widths and gaps from the Tran-Blaha functional: Comparison with many-body perturbation theory. Phys. Rev. B 2013, 87, 075121 10.1103/PhysRevB.87.075121.

[ref62] Chen S.; Walsh A.; Yang J.-H.; Gong X.-G.; Sun L.; Yang P.-X.; Chu J.-H.; Wei S.-H. Compositional dependence of structural and electronic properties of Cu 2 ZnSn (S, Se) 4 alloys for thin film solar cells. Phys. Rev. B 2011, 83, 125201 10.1103/PhysRevB.83.125201.

[ref63] Omata T.; Nagatani H.; Suzuki I.; Kita M. Wurtzite-derived ternary I–III–O2 semiconductors. Sci. Technol. Adv. Mater. 2015, 16, 024902 10.1088/1468-6996/16/2/024902. 27877769 PMC5036475

[ref64] Popescu V.; Zunger A. Effective band structure of random alloys. Physical review letters 2010, 104, 236403 10.1103/PhysRevLett.104.236403. 20867256

