# Supplementary material for: Electronic Properties of Zn2V(1–x)NbxN3 Alloys to Model Novel Materials for Light-Emitting Diodes
Source: J Phys Chem Lett. 2023 Oct 4;14(40):9118–25. doi: 10.1021/acs.jpclett.3c02242 (PMC10577778; doi:10.1021/acs.jpclett.3c02242)
Supplement: Supplementary file 1 — jz3c02242_si_001.pdf [file jz3c02242_si_001.pdf]

Supporting Information
of
Electronic Properties of $Zn_2V_{(1-x)}Nb_xN_3$ Alloys to Model Novel Materials for Light-Emitting Diodes

Ana-Maria Stratulat,[†] Christian Tantardini,[*,‡,¶] Maryam Azizi,[§] Tariq Altalhi,[‖] Sergey V. Levchenko,[†] and Boris I. Yakobson[*,¶,‖]

†*Skolkovo Institute of Science and Technology, Skolkovo Innovation Center, Bolshoy boulevard 30, Moscow, 143026, Russian Federation.*

‡*Hylleraas center, Department of Chemistry, UiT The Arctic University of Norway, PO Box 6050 Langnes, N-9037 Tromsø, Norway.*

¶*Department of Materials Science and NanoEngineering, Rice University, Houston, Texas 77005, United States of America.*

§*Université catholique de Louvain, Chemin des étoiles 8, bte L07.03.01, B-1348 Louvain-la-Neuve, Belgium*

‖*Chemistry Department, Taif University, Al Hawiyah, Taif 26571, Saudi Arabia*

E-mail: christiantantardini@ymail.com; biy@rice.edu

Computational Details

The valence band maximum (VBM) is found to be always located at the Γ-point of the first Brillouin zone. The direct band gap is therefore calculated at the Γ-point. The fundamental gap is calculated between the VBM at Γ-point and the conduction band minimum (CBM) at 0.00 0.25 0.00 k-point within the first Brillouin zone for all geometric configurations at different concentrations.

The orthorhombic primitive cell 1×1×1 coincides with the conventional cell with 24 atoms spanning on 7 compounds: one for ZnVN, one for $ZnV_{(0.75)}Nb_{(0.25)}N$, three for $ZnV_{(0.5)}Nb_{(0.5)}N$, one for $ZnV_{(0.25)}Nb_{(0.75)}N$, and one for ZnNbN. These atomic arrangements are visualized using the VESTA software[1] in Figure 4 of main text.

All configurations 1×1×2 and 1×2×1 orthorhombic supercells have 48-atoms.

The different nonequivalent arrangements of atoms in the 1 × 1 × 2 supercell span 40 different compounds: one for Zn_2VN_3, one for $Zn_2V_{(0.875)}Nb_{(0.125)}N_3$, five for $Zn_2V_{(0.75)}Nb_{(0.25)}N_3$, seven for $Zn_2V_{(0.625)}Nb_{(0.375)}N_3$, twelve for $Zn_2V_{(0.5)}Nb_{(0.5)}N_3$, seven for $Zn_2V_{(0.375)}Nb_{(0.625)}N_3$, five for $Zn_2V_{(0.25)}Nb_{(0.75)}N_3$, one for $Zn_2V_{(0.125)}Nb_{(0.875)}N_3$, and one for Zn_2NbN_3.

The different non-equivalent arrangements of atoms in the 1 × 2 × 1 supercell span 43 compounds: one for Zn_2VN_3, one for $Zn_2V_{(0.875)}Nb_{(0.125)}N_3$, six for $Zn_2V_{(0.75)}Nb_{(0.25)}N_3$, seven for $Zn_2V_{(0.625)}Nb_{(0.375)}N_3$, thirteen for $Zn_2V_{(0.5)}Nb_{(0.5)}N_3$, seven for $Zn_2V_{(0.375)}Nb_{(0.625)}N_3$, six for $Zn_2V_{(0.25)}Nb_{(0.75)}N_3$, one for $Zn_2V_{(0.125)}Nb_{(0.875)}N_3$, and one for Zn_2NbN_3.

All above-mentioned structures are fully optimized using DFT with PBE functional without spin-orbit coupling. The geometry optimization relies on Broyden-Fletcher-Goldfarb-Shanno (BFGS) algorithm,[2–5] with force tolerance for the maximum net force on atoms fixed at 10^{-6} Ha/bohr. The Γ-centered k-point meshes are: 2 × 4 × 4 for the primitive cell that generated a k-point grind in real space of length equal to 3.62380310E+01 Bohr; 2 × 4 × 2 for the 1 × 1 × 2 supercell; 2 × 2 × 4 for the 1 × 2 × 1 supercell. For convergence study we had Γ-centered k-point meshes: 1 × 2 × 2, 2 × 4 × 4, 4 × 8 × 8 and 6 × 12 × 12. For the primitive cell 1 × 1 × 1 convergence is realized for k-point mesh 2 × 4 × 4.

The computation of electronic band gap for these optimized structures, as well as for some other frozen-atom structures (see below) is performed with the TB09[6] exchange-correlation functional, as well as with the PBE density functional for reference.

The kinetic energy cutoff for all calculations is 40 Ha.

It is note of worthy, that the GW approximation[7,8] is a more reliable approach to the computation of band gaps. However, it is much more computationally demanding than semi-local DFT calculations, even in its non-self-consistent version G_0W_0. This initial screening allows us to demonstrate the reliability of Kohn-Sham (KS) band gap subsequently calculated for all possible configurations for the supercells 1×1×2 and 1×2×1.

Different pseudopotentials were used: projector augmented wave (PAW) pseudopotentials[9] for geometry optimization, electronic structure calculations for PBE, with 12 valence electrons for Zn, 13 for V, 13 for Nb and 5 valence electrons for N. The same number of valence electrons with exception of Zn (i.e., 2 valence electrons) was used for norm-conserving relativistic separable dual-space Gaussian pseudopotentials (HGH)[10] for electronic-structure calculations with TB09. All calculations are performed with Abinit version 9.6.2.[11–13]

ST. 1: Bader charges of V (Nb) as N for the Zn_2VN_3 (Zn_2NbN_3) primitive cell.

	V	Nb	N
Zn_2VN_3	1.80	-	-1.38
Zn_2NbN_3	-	2.24	-1.52

ST. 2: Fundamental and direct band gaps as a function of concentration calculated by PBE in optimized $Zn_2V_{(1-x)}Nb_xN_3$ alloy $1 \times 1 \times 1$ primitive cell.

x	Direct gap / eV	Fundamental gap / eV	Volume / $Bohr^3$	Energy total / eV
0.000	1.355	1.235	1913.194	-25128.197
0.250	1.451	1.366	1956.033	-24732.743
0.500	1.548	1.478	1997.894	-24337.252
0.500	1.616	1.549	1996.186	-24337.333
0.500	1.617	1.558	1996.630	-24337.334
0.750	1.747	1.700	2042.637	-23941.908
1.000	2.011	1.976	2084.673	-23546.526

ST. 3: Fundamental and direct band gaps as a function of concentration calculated by TB09 in optimized $Zn_2V_{(1-x)}Nb_xN_3$ alloy $1 \times 1 \times 1$ primitive cell.

x	Direct gap / eV	Fundamental gap / eV	Volume / $Bohr^3$	Energy total / eV
0.000	2.316	2.246	1913.194	-9606.039
0.250	2.367	2.324	1956.033	-9266.476
0.500	2.448	2.419	1997.894	-8926.817
0.500	2.533	2.510	1996.186	-8926.851
0.500	2.524	2.514	1996.630	-8926.856
0.750	2.630	2.626	2042.637	-8587.381
1.000	2.856	2.864	2084.673	-8247.649

ST. 4: Volume as a function of concentration in optimized $Zn_2V_{(1-x)}Nb_xN_3$ alloys $1 \times 2 \times 1$ supercells.

x	volume_atom / $bohr^3$	x	volume_atom / $bohr^3$
0.000	79.741	0.500	83.212
0.125	80.614	0.500	83.205
0.250	81.481	0.500	83.216
0.250	81.495	0.500	83.208
0.250	81.479	0.500	83.228
0.250	81.479	0.500	83.235
0.250	81.471	0.625	84.168
0.250	81.475	0.625	84.133
0.375	82.392	0.625	84.123
0.375	82.366	0.625	84.115
0.375	82.370	0.625	84.125
0.375	82.352	0.625	84.143
0.375	82.360	0.625	84.135
0.375	82.376	0.750	85.146
0.375	82.356	0.750	85.140
0.500	83.275	0.750	85.159
0.500	83.233	0.750	85.134
0.500	83.238	0.750	85.134
0.500	83.232	0.750	85.155
0.500	83.232	0.875	86.030
0.500	83.223	1.000	86.839
0.500	83.228		

ST. 5: Volume as a function of concentration in optimized $Zn_2V_{(1-x)}Nb_xN_3$ alloys $1 \times 1 \times 2$ supercells.

x	volume_atom / $bohr^3$	x	volume_atom / $bohr^3$
0.000	79.741	0.500	83.246
0.125	80.632	0.500	83.249
0.250	81.481	0.500	83.205
0.250	81.520	0.500	83.279
0.250	81.507	0.500	83.279
0.250	81.506	0.500	83.280
0.250	81.504	0.625	84.185
0.375	82.404	0.625	84.146
0.375	82.379	0.625	84.134
0.375	82.407	0.625	84.175
0.375	82.411	0.625	84.178
0.375	82.414	0.625	84.177
0.375	82.409	0.625	84.179
0.375	82.369	0.750	85.146
0.500	83.275	0.750	85.181
0.500	83.260	0.750	85.162
0.500	83.263	0.750	85.165
0.500	83.258	0.750	85.155
0.500	83.255	0.875	83.304
0.500	83.223	1.000	86.839

ST. 6: Direct band gaps and bowing parameter b as a function of concentration in optimized $Zn_2V_{(1-x)}Nb_xN_3$ alloy $1 \times 2 \times 1$ supercells.

x	TB09 / eV	b / eV	x	TB09 / eV	b / eV
0.000	2.247	-	0.500	2.521	0.123
0.125	2.284	0.357	0.500	2.528	0.094
0.250	2.336	0.339	0.500	2.397	0.618
0.250	2.264	0.720	0.500	2.521	0.120
0.250	2.336	0.338	0.500	2.447	0.418
0.250	2.311	0.473	0.500	2.363	0.752
0.250	2.303	0.515	0.625	2.485	0.607
0.250	2.342	0.307	0.625	2.566	0.264
0.375	2.360	0.491	0.625	2.560	0.290
0.375	2.396	0.336	0.625	2.549	0.334
0.375	2.315	0.683	0.625	2.585	0.180
0.375	2.385	0.385	0.625	2.470	0.673
0.375	2.410	0.278	0.625	2.446	0.773
0.375	2.343	0.563	0.750	2.628	0.402
0.375	2.400	0.321	0.750	2.630	0.390
0.500	2.419	0.528	0.750	2.504	1.065
0.500	2.424	0.509	0.750	2.621	0.438
0.500	2.453	0.393	0.750	2.627	0.408
0.500	2.430	0.487	0.750	2.633	0.376
0.500	2.442	0.436	0.875	2.710	0.638
0.500	2.520	0.124	1.000	2.856	-
0.500	2.420	0.527			

ST. 7: Fundamental band gaps and bowing parameter b as a function of concentration in optimized $Zn_2V_{(1-x)}Nb_xN_3$ alloy $1 \times 2 \times 1$ supercells.

x	TB09 / eV	b / eV	x	TB09 / eV	b / eV
0.000	2.217	-	0.500	2.504	0.131
0.125	2.272	0.231	0.500	2.507	0.117
0.250	2.320	0.300	0.500	2.397	0.558
0.250	2.264	0.600	0.500	2.505	0.125
0.250	2.318	0.314	0.500	2.439	0.391
0.250	2.306	0.375	0.500	2.363	0.692
0.250	2.300	0.410	0.625	2.482	0.573
0.250	2.317	0.318	0.625	2.560	0.239
0.375	2.353	0.443	0.625	2.553	0.269
0.375	2.387	0.297	0.625	2.548	0.290
0.375	2.315	0.602	0.625	2.567	0.209
0.375	2.375	0.348	0.625	2.470	0.625
0.375	2.398	0.250	0.625	2.446	0.725
0.375	2.340	0.498	0.750	2.623	0.387
0.375	2.387	0.295	0.750	2.626	0.374
0.500	2.411	0.501	0.750	2.503	1.027
0.500	2.424	0.449	0.750	2.617	0.422
0.500	2.442	0.379	0.750	2.619	0.408
0.500	2.430	0.427	0.750	2.633	0.336
0.500	2.429	0.428	0.875	2.707	0.633
0.500	2.508	0.114	1.000	2.856	-
0.500	2.420	0.467			

ST. 8: Direct band gaps as a function of concentration in optimized $Zn_2V_{(1-x)}Nb_xN_3$ alloy $1 \times 1 \times 2$ supercells.

x	TB09 / eV	b / eV	x	TB09 / eV	b / eV
0.000	2.276	-	0.500	2.492	0.294
0.125	2.317	0.294	0.5	2.498	0.269
0.250	2.357	0.342	0.500	2.530	0.143
0.250	2.335	0.458	0.500	2.486	0.318
0.250	2.364	0.307	0.500	2.468	0.391
0.250	2.333	0.472	0.500	2.454	0.445
0.250	2.354	0.360	0.625	2.513	0.533
0.375	2.395	0.422	0.625	2.572	0.281
0.375	2.417	0.325	0.625	2.575	0.267
0.375	2.415	0.333	0.625	2.548	0.384
0.375	2.397	0.413	0.625	2.530	0.460
0.375	2.413	0.341	0.625	2.548	0.385
0.375	2.393	0.430	0.625	2.537	0.433
0.375	2.432	0.260	0.750	2.630	0.427
0.500	2.446	0.477	0.750	2.594	0.621
0.500	2.468	0.390	0.750	2.642	0.365
0.500	2.462	0.416	0.750	2.626	0.449
0.500	2.476	0.358	0.750	2.634	0.407
0.500	2.458	0.431	0.875	2.826	-0.397
0.5	2.521	0.181	1.000	2.855	-

ST. 9: Fundamental band gaps as a function of concentration in optimized $Zn_2V_{(1-x)}Nb_xN_3$ alloy $1 \times 1 \times 2$ supercells.

x	TB09 / eV	b / eV	x	TB09 / eV	b / eV
0.000	2.215	-	0.500	2.487	0.192
0.125	2.297	-0.014	0.500	2.497	0.151
0.250	2.319	0.297	0.500	2.505	0.119
0.250	2.303	0.384	0.500	2.458	0.309
0.250	2.317	0.310	0.500	2.455	0.320
0.250	2.323	0.277	0.500	2.448	0.347
0.250	2.322	0.285	0.625	2.513	0.435
0.375	2.367	0.375	0.625	2.572	0.183
0.375	2.417	0.162	0.625	2.562	0.225
0.375	2.389	0.282	0.625	2.543	0.309
0.375	2.397	0.250	0.625	2.526	0.382
0.375	2.391	0.272	0.625	2.548	0.287
0.375	2.391	0.275	0.625	2.528	0.370
0.375	2.420	0.149	0.750	2.626	0.368
0.500	2.415	0.481	0.750	2.594	0.539
0.500	2.451	0.337	0.750	2.636	0.316
0.500	2.462	0.293	0.750	2.626	0.367
0.500	2.462	0.292	0.750	2.634	0.326
0.500	2.458	0.308	0.875	2.826	-0.467
0.500	2.509	0.103	1.000	2.855	-

ST. 10: Direct band gaps as a function of concentration in optimized $Zn_2V_{(1-x)}Nb_xN_3$ alloy $1 \times 2 \times 1$ supercells.

x	TB09 / eV	b / eV	x	TB09 / eV	b / eV
0.000	2.256	-	0.500	2.433	0.515
0.125	2.261	0.650	0.500	2.438	0.493
0.250	2.346	0.336	0.500	2.417	0.579
0.250	2.346	0.334	0.500	2.429	0.532
0.250	2.303	0.566	0.500	2.443	0.475
0.250	2.338	0.377	0.500	2.355	0.825
0.250	2.307	0.541	0.625	2.565	0.310
0.250	2.272	0.731	0.625	2.578	0.256
0.375	2.380	0.447	0.625	2.559	0.337
0.375	2.402	0.354	0.625	2.566	0.309
0.375	2.407	0.333	0.625	2.476	0.690
0.375	2.393	0.394	0.625	2.438	0.854
0.375	2.356	0.552	0.625	2.464	0.743
0.375	2.327	0.674	0.750	2.634	0.428
0.375	2.354	0.560	0.750	2.643	0.383
0.500	2.532	0.116	0.750	2.634	0.428
0.500	2.526	0.141	0.750	2.619	0.511
0.500	2.521	0.162	0.750	2.631	0.444
0.500	2.516	0.183	0.750	2.495	1.171
0.500	2.419	0.569	0.875	2.713	0.708
0.500	2.388	0.694	1.000	2.867	-
0.500	2.417	0.577			

ST. 11: Fundamental band gaps as a function of concentration in optimized $Zn_2V_{(1-x)}Nb_xN_3$ alloy $1 \times 2 \times 1$ supercells.

x	TB09 / eV	b / eV	x	TB09 / eV	b / eV
0.000	2.215	-	0.500	2.417	0.497
0.125	2.235	0.565	0.500	2.428	0.454
0.250	2.310	0.365	0.500	2.415	0.505
0.250	2.313	0.350	0.500	2.429	0.450
0.250	2.294	0.449	0.500	2.430	0.445
0.250	2.309	0.368	0.500	2.355	0.743
0.250	2.297	0.433	0.625	2.559	0.273
0.250	2.263	0.614	0.625	2.560	0.267
0.375	2.369	0.387	0.625	2.558	0.277
0.375	2.388	0.308	0.625	2.561	0.263
0.375	2.393	0.283	0.625	2.473	0.640
0.375	2.383	0.327	0.625	2.437	0.791
0.375	2.339	0.513	0.625	2.462	0.688
0.375	2.327	0.566	0.750	2.626	0.418
0.375	2.346	0.485	0.750	2.639	0.348
0.500	2.512	0.118	0.750	2.634	0.373
0.500	2.508	0.133	0.750	2.617	0.464
0.500	2.508	0.134	0.750	2.628	0.409
0.500	2.513	0.114	0.750	2.493	1.127
0.500	2.393	0.593	0.875	2.712	0.673
0.500	2.394	0.591	1.000	2.867	-
0.500	2.416	0.503			

ST. 12: Direct band gaps as a function of concentration in optimized $Zn_2V_{(1-x)}Nb_xN_3$ alloy $1 \times 1 \times 2$ supercells.

x	TB09 / eV	b / eV	x	TB09 / eV	b / eV
0.000	2.295	-	0.500	2.465	0.468
0.125	2.305	0.565	0.500	2.478	0.414
0.250	2.347	0.486	0.500	2.504	0.313
0.250	2.366	0.387	0.500	2.469	0.450
0.250	2.364	0.398	0.500	2.466	0.462
0.250	2.361	0.411	0.500	2.474	0.430
0.250	2.356	0.438	0.625	2.584	0.299
0.375	2.416	0.400	0.625	2.572	0.348
0.375	2.431	0.335	0.625	2.549	0.447
0.375	2.414	0.408	0.625	2.549	0.445
0.375	2.427	0.356	0.625	2.509	0.616
0.375	2.385	0.535	0.625	2.542	0.478
0.375	2.413	0.415	0.625	2.548	0.453
0.375	2.414	0.408	0.750	2.643	0.439
0.500	2.531	0.202	0.750	2.637	0.474
0.500	2.517	0.258	0.750	2.651	0.399
0.500	2.482	0.398	0.750	2.644	0.434
0.500	2.473	0.434	0.750	2.603	0.651
0.500	2.424	0.630	0.875	2.726	0.650
0.500	2.498	0.335	1.000	2.869	-

ST. 13: Fundamental band gaps as a function of concentration in optimized $Zn_2V_{(1-x)}Nb_xN_3$ alloy $1 \times 1 \times 2$ supercells.

x	TB09 / eV	b / eV	x	TB09 / eV	b / eV
0.000	2.222	-	0.500	2.465	0.322
0.125	2.277	0.231	0.500	2.478	0.269
0.250	2.320	0.340	0.500	2.504	0.167
0.250	2.321	0.332	0.500	2.469	0.305
0.250	2.351	0.172	0.500	2.466	0.317
0.250	2.318	0.351	0.500	2.456	0.357
0.250	2.331	0.282	0.625	2.584	0.183
0.375	2.416	0.206	0.625	2.572	0.232
0.375	2.429	0.152	0.625	2.549	0.331
0.375	2.413	0.220	0.625	2.549	0.329
0.375	2.427	0.162	0.625	2.509	0.500
0.375	2.372	0.396	0.625	2.542	0.361
0.375	2.401	0.270	0.625	2.548	0.337
0.375	2.404	0.257	0.750	2.643	0.343
0.500	2.512	0.135	0.750	2.637	0.377
0.500	2.514	0.125	0.750	2.651	0.302
0.500	2.471	0.298	0.750	2.644	0.337
0.500	2.473	0.289	0.750	2.603	0.554
0.500	2.398	0.589	0.875	2.726	0.567
0.500	2.498	0.189	1.000	2.869	-

ST. 14: Mixing energies $E_m = E_{total}(x) - (1-x) * E_{total}^{Zn_2VN_3} - (x) * E_{total}^{Zn_2NbN_3}$ at each concentration for $Zn_2V_{(1-x)}Nb_xN_3$ alloy $1 \times 1 \times 2$ supercells calculated with PBE functional, considering Zn $3d$-electrons as valence electrons.

x	E_m / eV	x	E_m / eV
0	-	0.5	-0.0036
0.125	-0.0004	0.5	-0.0032
0.25	-0.0023	0.5	-0.0050
0.25	-0.0019	0.5	-0.0022
0.25	-0.0011	0.5	-0.0020
0.25	-0.0016	0.5	-0.0019
0.25	-0.0006	0.625	-0.0038
0.375	-0.0035	0.625	-0.0052
0.375	-0.0043	0.625	-0.0058
0.375	-0.0023	0.625	-0.0051
0.375	-0.0028	0.625	-0.0048
0.375	-0.0031	0.625	-0.0044
0.375	-0.0032	0.625	-0.0051
0.375	-0.0035	0.75	-0.0148
0.5	-0.0014	0.75	-0.0141
0.5	-0.0011	0.75	-0.0150
0.5	-0.0013	0.75	-0.0152
0.5	-0.0010	0.75	-0.0153
0.5	-0.0011	0.875	0.3493
0.5	-0.0046	1	-

ST. 15: Mixing energies $E_m = E_{total}(x) - (1-x) * E_{total}^{Zn_2VN_3} - (x) * E_{total}^{Zn_2NbN_3}$ at each concentration for $Zn_2V_{(1-x)}Nb_xN_3$ alloy $1 \times 2 \times 1$ supercells calculated with PBE functional, considering Zn $3d$-electrons as valence electrons.

x	E_m / eV	x	E_m / eV
0	-	0.5	-0.0057
0.125	-0.0011	0.5	-0.0056
0.25	-0.0030	0.5	-0.0052
0.25	-0.0026	0.5	-0.0054
0.25	-0.0032	0.5	-0.0036
0.25	-0.0037	0.5	-0.0038
0.25	-0.0037	0.625	-0.0056
0.25	-0.0034	0.625	-0.0063
0.375	-0.0047	0.625	-0.0065
0.375	-0.0061	0.625	-0.0060
0.375	-0.0056	0.625	-0.0062
0.375	-0.0057	0.625	-0.0052
0.375	-0.0055	0.625	-0.0058
0.375	-0.0051	0.75	-0.0156
0.375	-0.0059	0.75	-0.0153
0.5	-0.0020	0.75	-0.0153
0.5	-0.0041	0.75	-0.0158
0.5	-0.0040	0.75	-0.0158
0.5	-0.0040	0.75	-0.0073
0.5	-0.0040	0.875	-0.0130
0.5	-0.0051	1	-
0.5	-0.0045		

ST. 16: Fixing volumes at each concentration configuration for Zn_2VN_3 alloy $1 \times 1 \times 1$ primitive cell calculated with TB09 functional.

x	Fundamental Band gap / eV	b
0.000	2.316	-
0.250	2.371	0.4480
0.500	2.466	0.5520
0.500	2.558	0.2120
0.500	2.557	0.2480
0.750	2.683	0.4853
1.000	2.865	-

ST. 17: Fixing volumes at each concentration configuration for $Zn_2V_0.5Nb_0.5N_3$ alloy $1 \times 1 \times 1$ primitive cell calculated with TB09 functional.

x	Fundamental Band gap / eV	b
0.000	2.184	-
0.250	2.314	0.2361
0.500	2.414	0.4710
0.500	2.533	-0.0050
0.500	2.512	0.0802
0.750	2.653	0.2839
1.000	2.880	-

ST. 18: Fixing volumes at each concentration configuration for Zn_2NbN_3 alloy $1 \times 1 \times 1$ primitive cell calculated with TB09 functional.

x	Fundamental Band gap / eV	b
0.000	2.117	-
0.250	2.255	0.2524
0.500	2.362	0.4994
0.500	2.444	0.1702
0.500	2.453	0.1330
0.750	2.608	0.3401
1.000	2.856	-

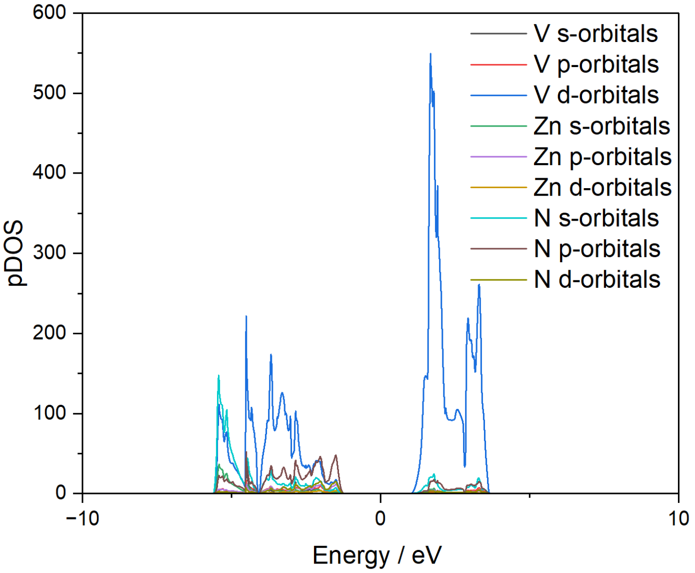

SF. 1: Projected density of states for the Zn_2VN_3 alloy.

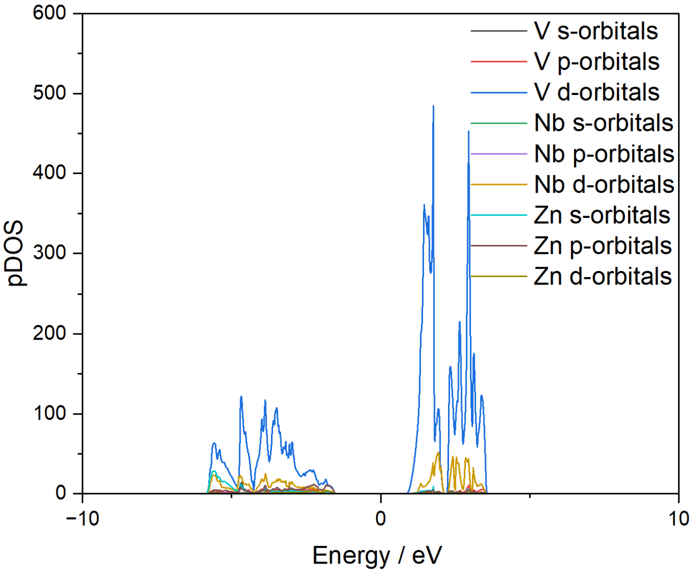

SF. 2: Projected density of states for the $Zn_2V_{0.75}Nb_{0.25}N_3$ alloy.

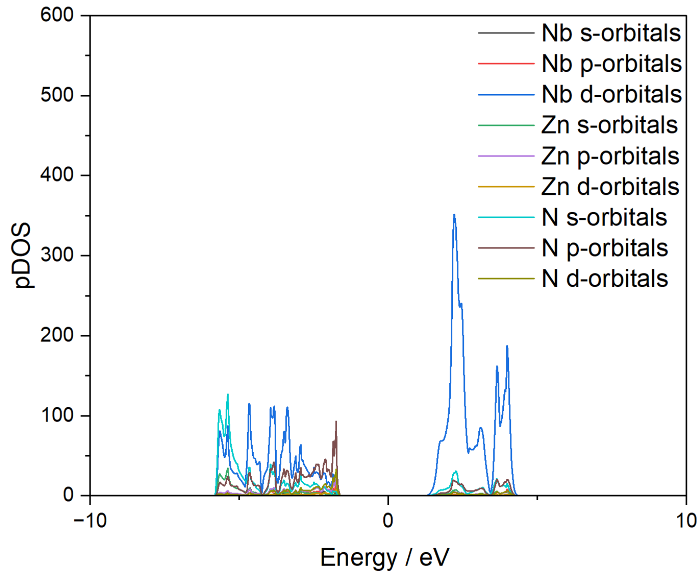

SF. 3: Projected density of states for the Zn_2NbN_3 alloy.

References

(1) Momma, K.; Izumi, F. VESTA 3 for three-dimensional visualization of crystal, volumetric and morphology data. *J. Appl. Crystallogr.* **2011**, *44*, 1272.

(2) Broyden, C. G. The Convergence of a Class of Double-Rank Minimization Algorithms 1. General Considerations. *IMA J. Appl. Math.* **1970**, *6 (1)*, 76–90.

(3) Goldfarb, D. A Family of Variable-Metric Methods Derived by Variational Means. *Math. Comp.* **1970**, *24 (109)*, 23–26.

(4) Shanno, D. F. Conditioning of Quasi-Newton Methods for Function Minimization. *Math. Comp.* **1970**, *24 (111)*, 647–656.

(5) Steihaug, T. *Practical Methods of Optimization Volume 1: Unconstrained Optimization*; Wiley, New York, 1980.

(6) Tran, F.; Blaha, P. Accurate Band Gaps of Semiconductors and Insulators with a Semilocal Exchange-Correlation Potential. *Phys. Rev. Lett.* **2009**, *102*, 226401.

(7) Hedin, L. New Method for Calculating the One-Particle Green's Function with Application to the Electron-Gas Problem. *Phys. Rev. A* **1965**, *139*, 795.

(8) Martin, R. M.; Reining, L.; Ceperley, D. *Interacting electrons*; Cambridge University Press, 2016.

(9) Jollet, F.; Torrent, M.; Holzwarth, N. A. Generation of Projector Augmented-Wave atomic data: A 71 element validated table in the XML format. *Computer Physics Communications* **2013**, *185*, 1246.

(10) Hartwigsen, C.; Goedecker, S.; Hutter, J. Relativistic separable dual-space Gaussian pseudopotentials from H to Rn. *Phys. Rev. B* **1998**, *58*, 3641.

(11) Gonze, X. et al. Recent developments in the ABINIT software package. *Computer Physics Communications* **2016**, *205*, 106–131.

(12) Gonze, X. et al. The Abinit project: Impact, environment and recent developments. *Computer Physics Communications* **2020**, *248*, 107042.

(13) Romero, A. H. et al. ABINIT: Overview, and focus on selected capabilities. *J. Chem. Phys.* **2020**, *152*, 124102.
